# Economic evaluation of a randomized controlled trial comparing mifepristone and misoprostol with misoprostol alone in the treatment of early pregnancy loss

**DOI:** 10.1371/journal.pone.0262894

**Published:** 2022-02-09

**Authors:** Charlotte C. Hamel, Marcus P. L. M. Snijders, Sjors F. P. J. Coppus, Frank P. H. A. Vandenbussche, Didi D. M. Braat, Eddy M. M. Adang

**Affiliations:** 1 Radboud University Medical Centre, Department of Obstetrics and Gynaecology, Nijmegen, GA, The Netherlands; 2 Department of Obstetrics and Gynaecology, Canisius-Wilhelmina Hospital, Nijmegen, GS, The Netherlands; 3 Maxima Medical Centre, Department of Obstetrics and Gynaecology, Veldhoven, DB, The Netherlands; 4 Department of Obstetrics and Gynaecology, Helios Klinikum Duisburg, Duisburg, Germany; 5 Department for Health Evidence, Radboud University Medical Centre, Nijmegen, GA, The Netherlands; Stellenbosch University, SOUTH AFRICA

## Abstract

**Background:**

In case of early pregnancy loss (EPL) women can either choose for expectant, medical or surgical management. One week of expectant management is known to lead to spontaneous abortion in approximately 50% of women. Medical treatment with misoprostol is known to be safe and less costly than surgical management, however less effective in reaching complete evacuation of the uterus. Recently, a number of trials showed that prompt treatment with the sequential combination of mifepristone with misoprostol is superior to misoprostol alone in reaching complete evacuation. In this analysis we evaluate whether the sequential combination of mifepristone with misoprostol is cost-effective compared to misoprostol alone, in the treatment of EPL.

**Methods and findings:**

A cost-effectiveness analysis (CEA) from a healthcare perspective was performed alongside a randomised controlled trial (RCT) in which standard treatment with misoprostol only was compared with a combination of mifepristone and misoprostol, in women with EPL after a minimum of one week of unsuccessful management. A limited societal perspective scenario was added. This RCT, the Triple M trial, was a multicentre, randomized, double-blinded, placebo-controlled trial executed at 17 hospitals in the Netherlands. The trial started on June 27^th^ 2018, and ended prematurely in January 2020 due to highly significant outcomes from the predefined interim-analysis. We included 351 women with a diagnosis of EPL between 6 and 14 weeks gestation after at least one week of unsuccessful expectant management. They were randomized between double blinded pre-treatment with oral mifepristone 600mg (N = 175) or placebo (N = 176) taken on day one, both followed by misoprostol orally. In both groups, an intention-to-treat analysis was performed for 172 patients, showing a significant difference in success rates between participants treated with mifepristone and misoprostol versus those treated with misoprostol alone (79.1% vs 58.7% respectively). In this cost-effective analysis we measured the direct, medical costs related to treatment (planned and unplanned hospital visits, medication, additional treatment) and indirect costs based on the IMTA Productivity Cost Questionnaire (iPCQ). Quality Adjusted Life Years (QALY’s) were calculated from participants’ scores on the SF-36 questionnaires sent digitally at treatment start, and one, two and six weeks later. We found medical treatment with placebo followed by misoprostol to be 26% more expensive compared to mifepristone followed by misoprostol (p = 0.001). Mean average medical costs per patient were significantly lower in the mifepristone group compared to the placebo group (€528.95 ± 328.93 vs €663.77 ± 456.03, respectively; absolute difference €134.82, 95% CI 50,46–219,18, p = 0.002). Both indirect costs and QALY’s were similar between both groups.

**Conclusion:**

The sequential combination of mifepristone with misoprostol is cost-effective compared with misoprostol alone, for treatment of EPL after a minimum of one week of unsuccessful expectant management.

## Introduction

EPL is defined as a non-viable first trimester intra-uterine pregnancy, in which there may be an anembryonic gestation or embryonic death [1, 2]. It is the most common complication in early pregnancy, with a reported incidence varying from 10% to 28% of pregnancies [3, 4]. The estimated annual number of pregnancies worldwide is 227 million, meaning every year millions of women will seek treatment for EPL.

Three treatment options exist for EPL: expectant, surgical or medical management. In many European countries, including the Netherlands, expectant management of miscarriage (waiting for the miscarriage to occur spontaneously) for at least one week is common practice, as spontaneous complete evacuation occurs in up to 50% of women [5, 6]. However, after this period of expectant management, around half of women experiencing EPL may require treatment. Although very successful in reaching complete evacuation, surgical management, i.e., uterine aspiration, is associated with risks of early and late complications, such as adhesion formation and increased risk of premature delivery in subsequent pregnancies, and higher costs [7–9].

Multiple international guidelines recommend prostaglandins as primary medical treatment of EPL [2, 10]. Misoprostol tablets, a prostaglandin E1 analogue are, although off-label, widely used, relatively cheap, easy to apply and proven safe, not requiring special storage or temperature conditions [2, 10, 11] Medical management using misoprostol without previous expectant management may result in success rates of 66.0% to 88.5% [12, 13]. After one week of unsuccessful expectant management, the success rate of misoprostol treatment drops to around 54% [14, 15]. In short, surgical treatment is associated with risks and higher costs, but medical treatment with misoprostol is limited in terms of efficacy.

Recently, whether or not preceded by expectant management, the sequential combination of mifepristone and misoprostol has been shown to be more successful in reaching complete expulsion than misoprostol alone in case of EPL as proven by both our and other research groups [16–18].

Here, we present a cost-effectiveness analysis (CEA), performed as a secondary analysis alongside the Triple M Trial, investigating the sequential combination of mifepristone and misoprostol versus placebo followed by misoprostol after at least one week of unsuccessful expectant management in women with EPL.

## Materials and methods

This economic evaluation was performed alongside the Triple M Trial, a nationwide multicentre double-blinded placebo-controlled RCT conducted in 17 Dutch hospitals [16]. A CONSORT checklist and further details about study design, sample size calculation, study procedures and outcome have been described previously [19].

In short, women of at least 16 years of age with confirmed EPL by ultrasonography (at gestational age 6 to 14 weeks), managed expectantly for at least one week without aborting spontaneously, were eligible for inclusion. After written informed consent, women were randomly assigned to either mifepristone 600 mg orally or an identical appearing placebo, containing no active ingredients, followed 36–48 hours later by misoprostol 400 μg 2dd on day three and, if necessary, again on day four.

Successful treatment was defined as ultrasonographic confirmed expulsion of the gestational sac and an endometrial thickness <15 mm after a maximum of 6 weeks after treatment, using only the allocated therapy.

Both the mifepristone and identical appearing placebo tablets were purchased from the same manufacturer, Exelgyn (Groupe Nordic Pharma, France). Exelgyn had no further role in the design or execution of the study, nor in the analysis of study results.

### Ethical approval

Ethical approval for the Triple M Trial was obtained from the Medical Research Ethical Committee region Arnhem-Nijmegen and the National Central Committee on Research involving Human Subjects (file number NL 62449.091.17) In addition, the board of directors of each of the participating centres gave approval to conduct this trial on their respective locations.

### Economic evaluation

Cost-effectiveness was evaluated from a health care perspective. In an additional scenario cost-effectiveness was determined from a societal perspective. Costs, usually skewed, were analysed by a generalized linear model with a log link relating the conditional mean to the treatment dummy using a gamma distribution specifying the relationship between the variance and the mean. The choice for a log link-based model, besides flexibility, was mainly made to present cost difference as a percentage. By doing so, the result is more generalizable to other countries with different healthcare cost systems, as absolute cost figures might differ, relative cost figures may be more insightful.

Absolute cost figures are also presented. Unit costs for outpatient visits, ultrasonography, hospital admission, hysteroscopy, uterine aspiration and packed cells were provided by the financial department of the Radboud University Medical Centre, Nijmegen, the Netherlands. Costs for medication were derived from the Dutch Formulary for medication (https://www. therapeutischkompas.nl (accessed 8 April 2020)), to deliver costs in daily practice. All costs are expressed in Euros, direct costing data were available from all 344 participants.

Indirect costing data about productivity loss were obtained by means of the IMTA Productivity Cost Questionnaire (iPCQ).

Health related quality of life was based on the participants scores on the Dutch version of the RAND-36 questionnaires, measured at four moments: at treatment start, after one, two and six weeks. If the questionnaire was not started at baseline, or the next three questionnaires were not or only barely completed (<20%), these participants were excluded from the quality-of-life analysis. For the remaining questionnaires, missing data were imputed using the ‘last observation carried forward’ (LOCF) method, a conservative method of data imputation when outcomes are expected to improve over time [20], as expected in these medical circumstances.

Then, according to the Short Form Six Dimension (SF-6D) health state classification form, a preference-based index was deduced [21]. Quality adjusted life years (QALYs) were thus calculated over the period of evaluation using the SF-6D index scores (utilities) multiplied by the consecutive time periods applying the trapezium method. This method evaluates the area under the curves by dividing the total area into smaller trapezoids rather than using rectangles, with the additional advantage of applying correction at each moment of measurement [22].

Incremental cost-effectiveness ratios (ICERs) were calculated generating 95% CIs from 1000 bootstrapped replications with replacement to test the robustness of our cost-effectiveness result. These results are presented in a Cost-Effectiveness-plane (CE plane), graphically illustrating costs and effects of an intervention, and in a Cost-Effectiveness Acceptability Curve (CEAC) summarizing the impact of uncertainty on the economic evaluation.

## Results

### Participants

**Fig 1:**
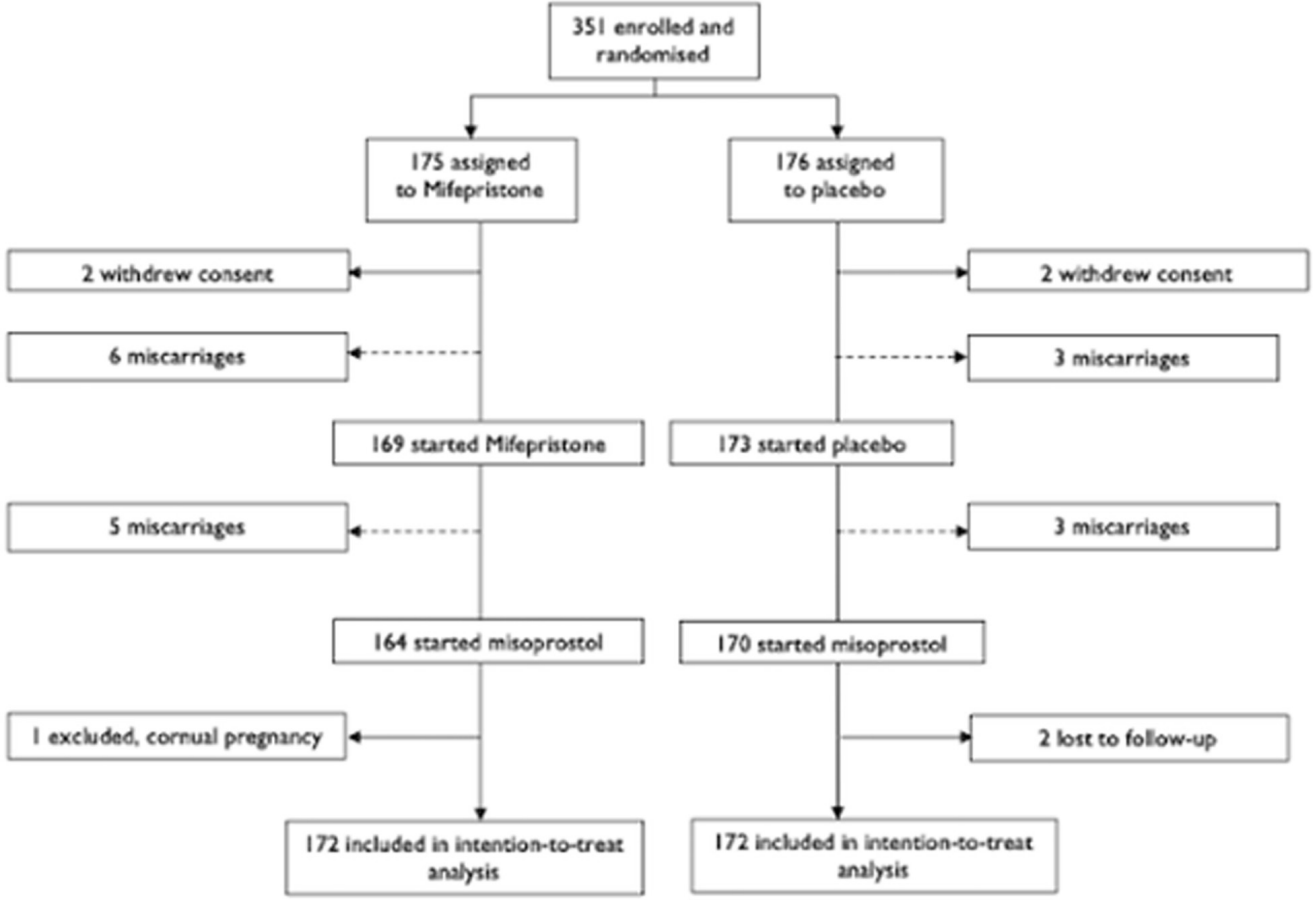
Trial profile. ⇢ = included in intention-to-treat analysis. → = excluded from intention-to-treat analysis.

Between June 27^th^ 2018 and January 8^th^ 2020, a total of 351 patients were enrolled and randomized to either pre-treatment with mifepristone (175 patients) or placebo (176 patients) prior to misoprostol after at least one week of unsuccessful expectant management. Based on the superiority of one of the treatments, the study was halted prematurely, as advised by the trials Data Safety Monitoring Board. After excluding patients who either withdrew their consent, were lost to follow-up or in retrospect did not meet inclusion criteria (one patient turned out to have a cornual pregnancy) 172 participants remained in each group, see Fig 1.

### Clinical effectiveness and QALYs outcomes

Baseline characteristics were comparable between the two groups, as shown in Table 1. The percentage of patients reaching complete evacuation of the uterus without additional treatment (the primary outcome) was significantly higher in the mifepristone group than in the placebo group (79.1% and 58.7% respectively), p = 0.000. Additionally, the number of uterine aspirations performed to achieve complete evacuation was significantly lower in the mifepristone group (19/172 participants) than in the placebo group (51/172 participants), p = 0.000, see also Table 1.

**Table 1:** Basis characteristics and clinical outcomes.

Characteristic	Mifepristone and Misoprostol N = 172	Placebo and Misoprostol N = 172
Age (years)		
Mean (SD)	32.95 (4.39)	32.69 (4.30)
BMI		
Mean (SD)	24.70 (4.44)	24.08 (3.84)
Unknown	28	34
Race or ethnic group		
Caucasian	156 (90.7%)	155 (90.1%)
Other	12 (7.0%)	13 (7.6%)
Unknown	4 (2.3%)	4 (2.3%)
Gravidity		
1	60 (34.9%)	75 (43.6%)
2	63 (36.6%)	53 (30.8%)
≥3	49 (28.5%)	44 (25.6%)
Parity		
0	83 (48.3%)	94 (54.7%)
1	70 (40.7%)	64 (37.2%)
≥2	19 (11.0%)	14 (8.1%)
Gestational age based on amenorrhoea (days)		
Mean (SD)	71.22 (11.03)	70.09 (11.57)
Unknown	3	3
Diagnosis		
Embryo without cardiac activity	123 (71.5%)	115 (66.9%)
Anembryonic gestation	49 (28.5%)	57 (33.1%)
Prior miscarriage	51 (29.7%)	52 (30.2%)
Of these: misoprostol treatment for prior miscarriage	13 (25.5%)	19 (36.5%)
Of these: successful misoprostol treatment	7 (53.8%)	12 (63.2%)
**Clinical outcomes**		
Complete evacuation	136 (79.1%)	101 (58.7%)
Uterine aspiration	19 (11.0%)	51 (29.7%)
Other additional therapy	17 (9.9%)	20 (11.6%)

The RAND-36 questionnaire scores were available from 182 participants (94 in the mifepristone and 88 in the placebo group), with again no significant differences in the basic characteristics. These scores were analysed, showing that SF-6D utility index scores did not differ significantly between groups on either measuring moment, see Table 2. The mifepristone group had, over the six weeks period of evaluation, a mean QALY of 0.0853 (95% CI 0.0820–0.0890) compared to a mean QALY of 0.0860 (95% CI 0.0833–0.0892) in the placebo group (p = 0.775).

**Table 2:** SF-6D utility index scores per measuring moment in 1000 bootstrapped simulations.

	Mean utility scores (± SD)		
	Mifepristone and misoprostol (N = 94)	Placebo and misoprostol (N = 88)	P-value
**Measuring moment**			
Treatment start (T = 1)	0.7387 (± 0.097)	0.7346 (± 0.109)	0.783
One week after treatment start (T = 2)	0.6713 (± 0.109)	0.6938 (± 0.119)	0.187
Two weeks after treatment start (T = 3)	0.6852 (± 0.102)	0.6979 (± 0.115)	0.420
Six weeks after treatment start (T = 4)	0.7416 (± 0.119)	0.7609 (± 0.106)	0.232

### Costs and cost-effectiveness

Cost analysis (health care perspective) showed that the mean (± SD) direct, medical costs were significantly lower in the mifepristone group compared to the placebo group (Table 3: €528.95 ± 328.93 vs €663.77 ± 456.03, respectively; absolute difference €134.82, 95% CI 50,46–219,18, p = 0.002). Medical treatment with placebo followed by misoprostol, analysed by the log link-based model, is 26% more expensive compared to mifepristone followed by misoprostol (p = 0.001).This shows that the overall cost reduction achieved by pre-treatment with mifepristone outweighs the costs of mifepristone tablets.

**Table 3:** Direct costs for completed treatment per patient.

	Mean direct, medical costs per patient (±SD); €			
	Mifepristone and misoprostol (N = 172)	Placebo and misoprostol (N = 172)	Absolute between-group difference (95% CI)	P-value*
***Direct*, *medical costs***	***528*.*95 (± 328*.*93)***	***663*.*77 (± 456*.*03)***	***134*.*82 (50*.*46–219*.*18)***	***0*.*002***
Medication	71.27 (± 8.95)	37.76 (± 11.91)	33.50 (35.73–31.27)	**0.000**
Ultrasound	150.12 (± 50.32)	151.14 (± 45.63)	1.02 (-9.17–11.21)	0.844
Hospital visits	179.35 (± 12.93)	179.44 (± 12.23)	0.09 (-2.58–2.76)	0.946
Hospital admissions	46.28 (± 136.20)	104.13 (± 179.70)	57.85 (24.03–91.68)	**0.001**
Uterine aspiration	64.92 (± 184.76)	174.26 (± 269.19)	109.34 (60.35–158.33)	**0.000**
Hysteroscopy	16.94 (± 64.99)	13.86 (± 59.15)	3.08 (-16.26–10.10)	0.646
Packed cells	0 (± 0)	3.10 (± 40.66)	3.10 (-3.02–9.22)	0.319
Antibiotics	0.079 (± 1.04)	0.079 (± 1.04)	0.00 (-0.22–0.22)	1.000

Plus-minus values are mean ± SD.

*Student’s t-test; a P-value <0.05 was considered statistically significant.

Total indirect costs from productivity loss, based on scores of the fully completed IPCQ questionnaire were available for 116 participants, 59 in the mifepristone and 57 in the placebo group. Basic characteristics were again similar between these groups. Mean (± SD) indirect costs were comparable between both groups, being €2155.59 ± 3371.29 in the mifepristone group, compared to €2161.70 ± 3100.94 in the placebo group (p = 0.992) and consequently did not alter the cost-effectiveness outcome.

**Fig 2:**
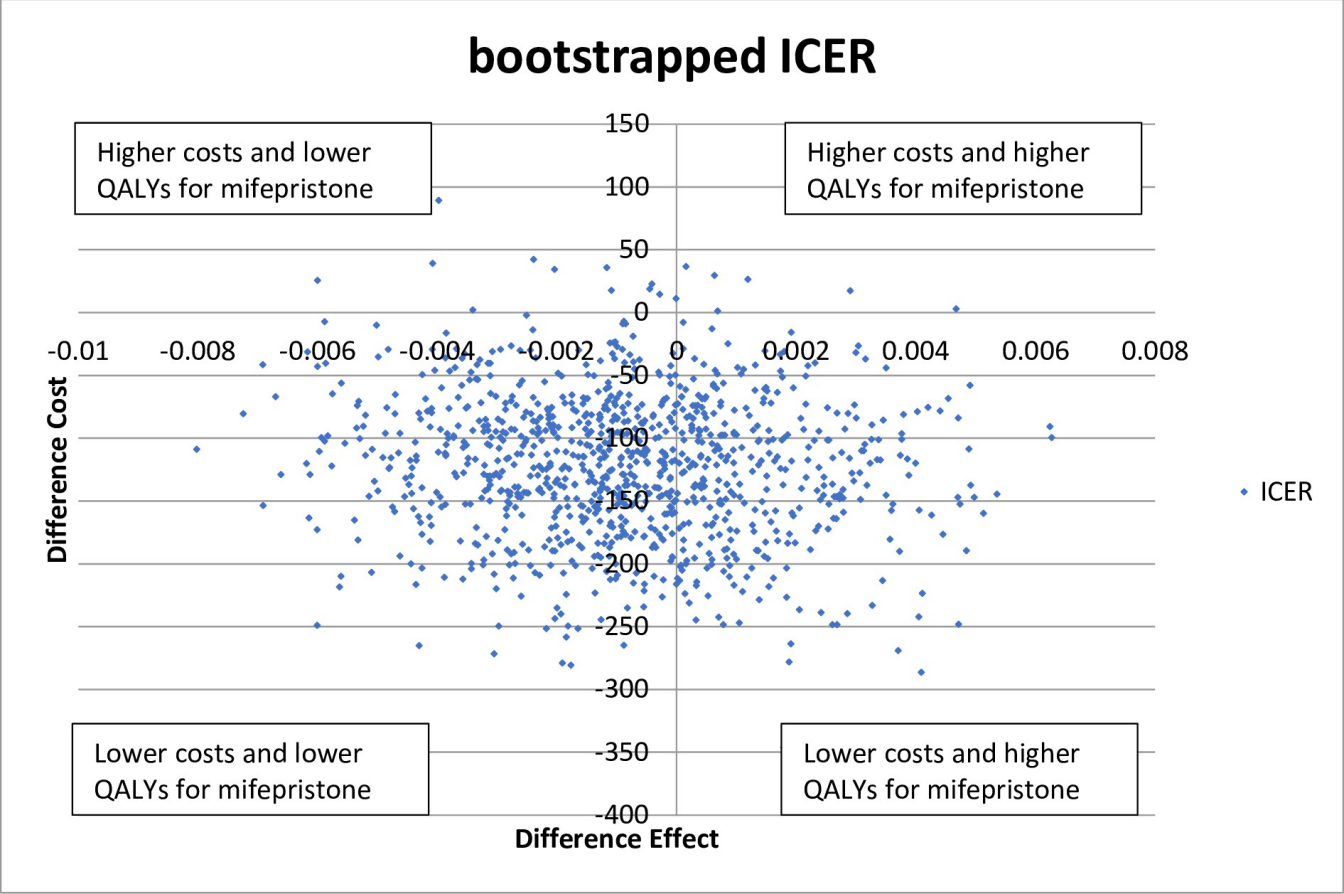
Cost-effectiveness plane: Scatter plot showing the mean difference in cost per QALY gained i.e. incremental cost-effectiveness, in 1000 bootstrapped simulations, (standard strategy in origin).

The bootstrapped Incremental Cost-effectiveness ratios (ICERs), or costs per QALY gained, are plotted on the cost-effectiveness (CE) plane in Fig 2, with the control strategy in the origin. This shows that the experimental strategy, i.e. pre-treatment with mifepristone prior to misoprostol, is less costly compared to the standard strategy, i.e. misoprostol only, illustrated by a negative difference in costs. Regarding QALY’s the effect is equal for both strategies (differences in effect are not mainly positive nor negative). As the experimental strategy is cheaper but equally effective, it is therefore cost-effective.

**Fig 3:**
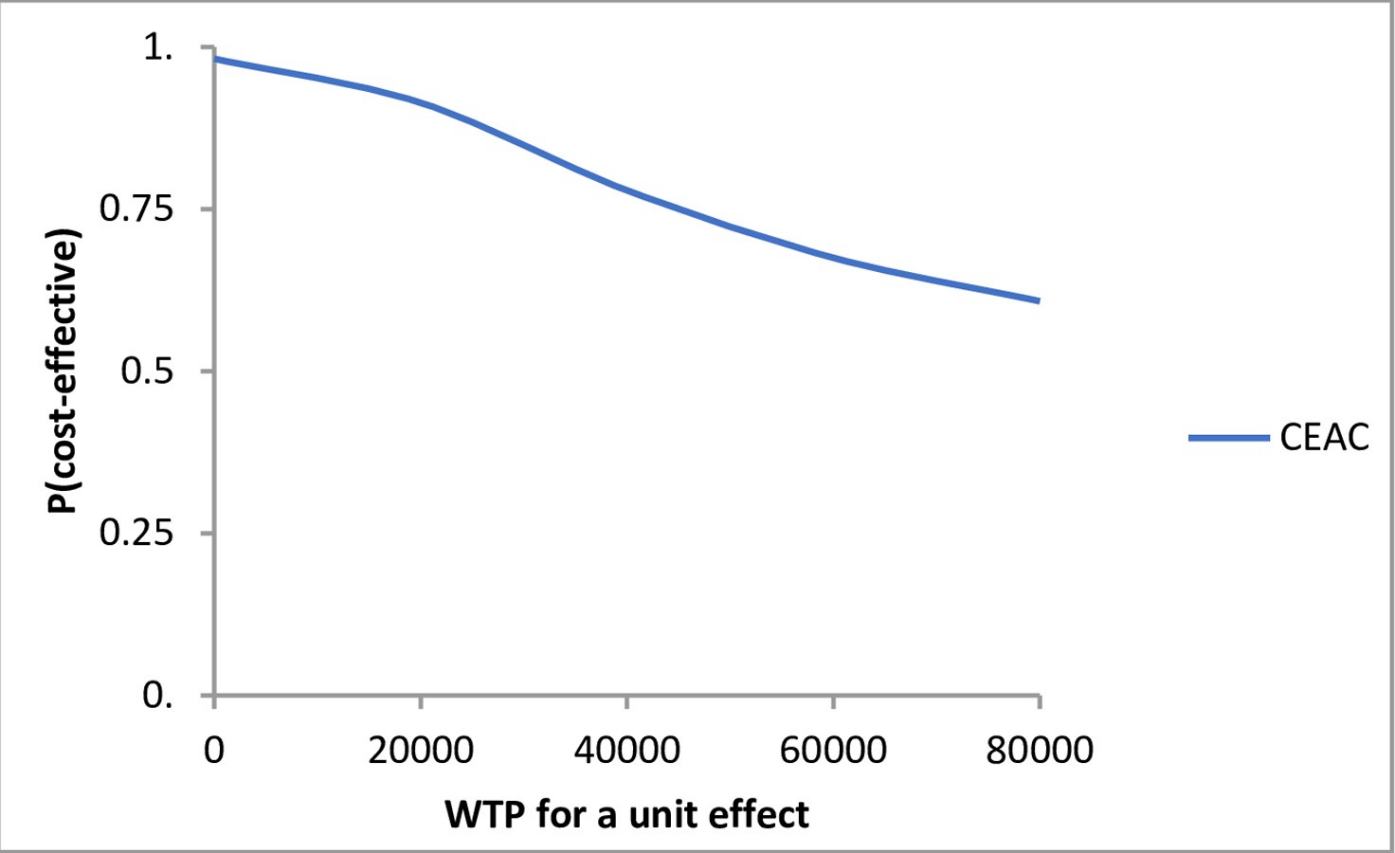
Cost effectiveness acceptability curve.

From Fig 2 a cost-effectiveness acceptability curve (CEAC) can be deduced, illustrated in Fig 3. The probability that the experimental strategy is cost-effective, compared to the control, decreases if the willingness to pay (WTP) increases, due to a small but insignificant difference between the point estimates of the SF-6D score in the control and experimental group.

Over the relevant range, with a maximum of €80.000 as additional costs per QALY, the probability that the experimental strategy is more cost-effective than the control strategy is higher.

**Fig 4:**
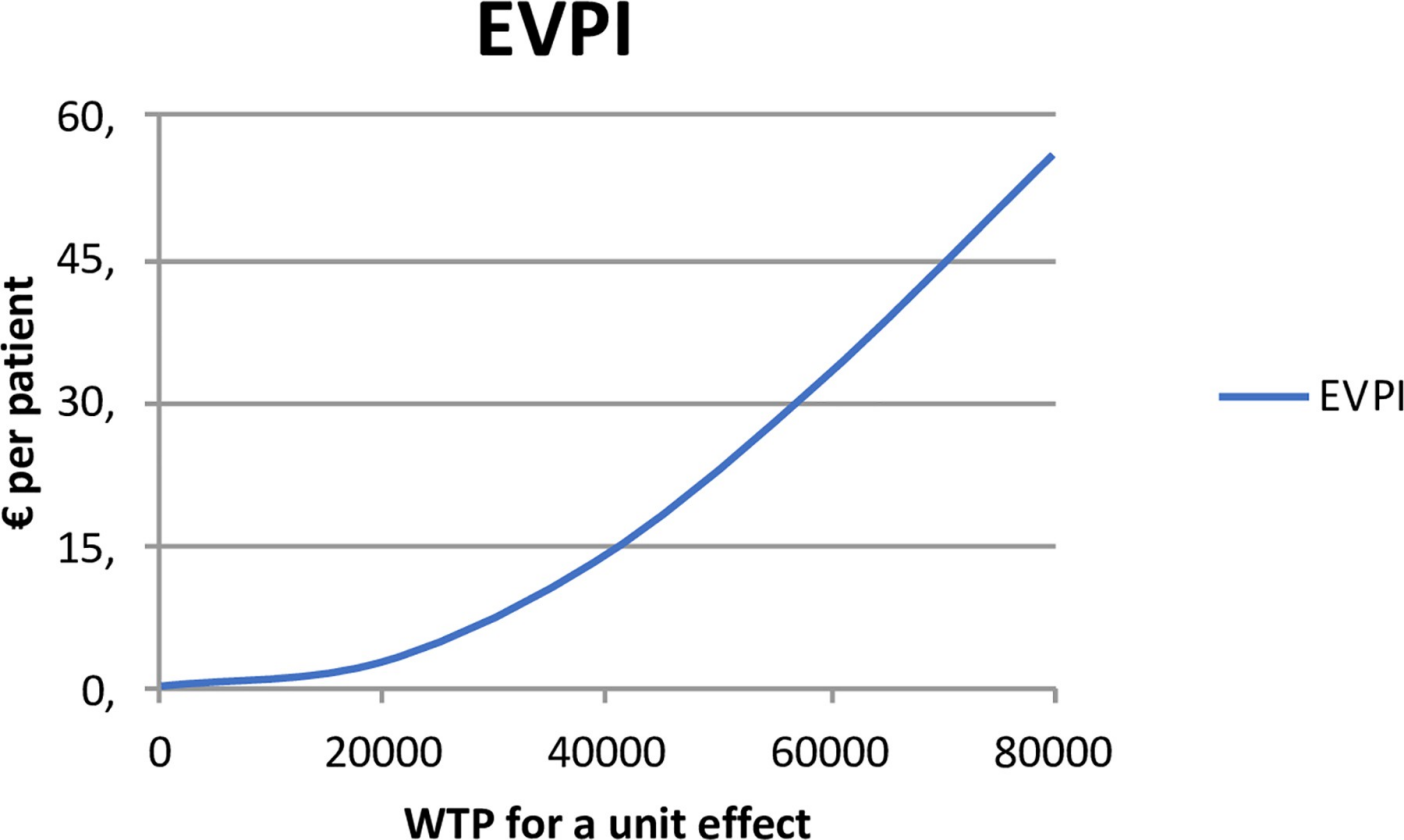
Expected value of perfect information curve.

Uncertainty of these probabilities increases when the WTP for a QALY increases. This is shown in Fig 4; with the EVPI (Expected Value of Perfect Information, the price that one would be willing to pay in order to gain access to perfect information) increasing with increasing WTP. This shows that when the WTP increases, perfect information becomes more valuable (i.e. the EVPI rises), to counter the increased uncertainty. At a WTP of €80.000, the EVPI is about €55 per patient. On the other hand, if one only focuses on cost the EVPI is near €0, as cost are significantly lower in the experimental group, making the uncertainty surrounding the cost driven cost-effectiveness decision rule vanish.

## Discussion

This cost-effectiveness analysis shows that, after one week of unsuccessful expectant management, pre-treatment with mifepristone 600 mg taken orally, prior to misoprostol 400 μg 2dd taken orally, for one or two days, is a cost-effective alternative to misoprostol alone as medical management for EPL. Overall, treatment with misoprostol only was found to be 26% more expensive compared to treatment with mifepristone and misoprostol (experimental strategy, p = 0.001). From a societal perspective, indirect costs were comparable between both groups. Although the experimental treatment has slightly higher medication costs, the increase in clinical effectiveness results in a less expensive treatment regimen overall, with a difference of € 134,80.

The key strength of this cost-effectiveness analysis is that it was performed alongside a well-designed RCT, enabling firm conclusions. Additionally, as we opted for a generalized linear model with a log link, comparison with other cost-effectiveness analyses on this treatment regimen is facilitated. A limitation might be that QALYS and indirect costs could not be calculated for all participants, as not all participants completed the questionnaires. However, with a response rate of 66% we believe solid conclusions can be drawn from these data. Furthermore, it is important to realize that LOCF as an imputation method, which was used here, can be prone to bias, depending on the distribution of the observed values. However, as the pattern over time in the experimental and control group is comparable before and after imputation, and the trend observed after LOCF is similar compared to that in the pilot study of our RCT, this imputation method appears suitable for this analysis.

Recently Nagendra et al. [23], reported on a cost-effectiveness analysis, based on a pragmatic comparative effectiveness trial in the United States. In this trial, prompt treatment, without expectant management in case of EPL, was applied. Treatment consisted of 200 mg mifepristone followed by 800 mcg misoprostol vaginally, or 800 mcg misoprostol vaginally only, without prior use of a placebo. Successful treatment was defined as loss of the gestational sac at 24 to 96 hours after misoprostol use, and no need for surgical intervention in the following 30 days. Nagendra et al. also concluded that pre-treatment with mifepristone is the cost-effective alternative in case of EPL. This was also the conclusion of a secondary analysis of their data, in which Monte Carlo simulations were used to assess contribution of different expense categories [24]. An in-depth comparison between these two trials may be difficult as study design varies substantially, regarding medication regimen, applying expectant management prior to inclusion or prompt treatment and mean gestational age, which is approximately three weeks higher in our study. Of course, the healthcare system is arranged quite different in the United States compared to the Netherlands, especially regarding costs.

Comparing both studies, direct costs were significantly lower in the mifepristone group compared with the placebo group in this Triple M trial. In contrast to Nagendra et al., uterine aspiration, hospital admission as well as pharmaceutical costs were all significantly different between both treatment groups in our analysis. A significantly higher number of uterine aspirations, automatically involving hospital admission in the Netherlands, led to higher costs for the misoprostol only group in this study. This may be partly due to the fact that in the Netherlands uterine evacuation is usually performed in a clinical setting, accompanied by hospital admission, whereas in the United states manual vacuum aspiration (MVA) in an outpatient setting may be more common, thus being potentially cheaper [25, 26]. Regarding pharmaceutical costs, in two other studies showing a therapeutical advantage of prompt pre-treatment with mifepristone in case of EPL a dosage of 200 mg mifepristone was used, potentially leading to less direct pharmaceutical costs [17, 18]. In our RCT after at least one week of unsuccessful expectant management a dosage of 600 mg mifepristone was chosen for three reasons. Firstly, non-viable pregnancies may require a higher dosage of mifepristone then is used in medical abortion. Secondly, as these pregnancies still remain intra-uterine, even after a minimum of one week of expectant management, a higher dose may be needed. In our Triple M Trial, the average gestational age Thirdly, a higher dosage of mifepristone does not necessarily lead to more side-effects, and has even been found to cause less pain in case of medical abortion of viable first-trimester pregnancy [27].

As the patent of mifepristone has expired, pharmaceutical costs are more and more reduced. In fact, during this Triple M trial, costs for Mifepristone were €11,99 for one tablet of 200 mg, compared to reported $90,—in the PreFair trial by Schreiber et al. Future dosage finding studies for mifepristone as well as misoprostol may provide definitive clarity about optimal dosage for efficacy, regarding prior expectant management yes or no, adverse reactions, quality of life, and costs.

Further comparison of both CEA’s reveals that, although the maximum WTP is $150,000 for the United States [28], against €80.000 (approximately $94,000) in the Netherlands [29], both CEA’s conclude that over the relevant range (for the country involved where each trial was performed), the probability that mifepristone pre-treatment is cost-effective is highest.

In the Triple M Trial expectant management around EPL treatment was deliberately chosen as a preferred policy. Apart from a minimum of one week of expectant management, we asked participants, taking their preference into account, to return six weeks after treatment start if ultrasound at the first follow-up at two weeks was suspect for retained products of conception (RPOCs) i.e. gestational sac expulsed, but endometrial thickness > 15 mm [30]. This posttreatment expectant policy is based on three pillars. Firstly, it has been shown that an expectant policy in case of suspected RPOC after misoprostol treatment for EPL is equally safe compared to prompt uterine aspiration, thus preventing additional treatment in the majority (up to 85%) of women [31]. Secondly, uterine evacuation has not been proven cost-effective compared with expectant management in this scenario [32]. Finally, it has been shown that, in women in whom expectant management was applied in case of suspected RPOC after misoprostol treatment for EPL, health-related quality of life improved more and earlier than of women who underwent surgical evacuation [33]. This preferred expectant policy and thus restricted use of additional therapy may lead to less interventions, resulting in lower costs, without negatively influencing health-related quality of life and also crucial, accepting patient’s preference. However, we realize that in other settings prompt treatment may be preferred, as expectant management can only be applied under the condition that a solid and easily accessible (24/7) backup setting for emergencies is available. This may not be the case in all healthcare systems worldwide, thus influencing feasibility and cost effectiveness.

In conclusion, pre-treatment with mifepristone prior to misoprostol in case of EPL after a minimum of one week of unsuccessful expectant management not only increases success-rates, but is also cost-effective compared to misoprostol alone. This will have important implications worldwide as both mifepristone and misoprostol, not requiring special storage conditions, become more and more freely available and affordable in terms of pharmaceutical costs. These features, combined with an overall lower risk of complications argue for a more widespread use of, pre-treatment with mifepristone in case of EPL whether or not after a period of expectant management in both high- and low-income countries. Future dose-finding studies might provide definitive clarity about the optimal dosage of mifepristone, in which costs should also be taken into account.

## References

[1] Neilson JP, Gyte GML, Hickey M, Vazquez JC, Dou L. Medical treatments for incomplete miscarriage (less than 24 weeks). Chichester, UK: John Wiley & Sons, Ltd; 2009. Available from: www.thecochranelibrary.com.

[2] NICE guideline. Ectopic pregnancy and miscarriage: diagnosis and initial management. Natl Inst Heal Care Excell [Internet]. 2019;(April). Available from: https://www.nice.org.uk/guidance/ng126/chapter/Recommendations#symptoms-and-signs-of-ectopic-pregnancy-and-initial-assessment.

[3] Ammon Avalos L, Galindo C, Li DK. A systematic review to calculate background miscarriage rates using life table analysis. Birth Defects Res Part A—Clin Mol Teratol. 2012;94(6):417–23. doi: 10.1002/bdra.23014 22511535

[4] Buck Louis GM, Sapra KJ, Schisterman EF, Lynch CD, Maisog JM, Grantz KL, et al. Lifestyle and pregnancy loss in a contemporary cohort of women recruited before conception: The LIFE Study. Fertil Steril. 2016;106(1):180–8. doi: 10.1016/j.fertnstert.2016.03.009 27016456 PMC4930884

[5] Luise C, Jermy K, May C, Costello G, Collins WP, Bourne TH. Outcome of expectant management of spontaneous first trimester miscarriage: observational study. BMJ [Internet]. 2002;324(7342):873–5. Available from: http://www.pubmedcentral.nih.gov/articlerender.fcgi?artid=101397&tool=pmcentrez&rendertype=abstract. doi: 10.1136/bmj.324.7342.873 11950733 PMC101397

[6] Wieringa-de Waard M, Vos J, Bonsel GJ, Bindels PJE, Ankum WM. Management of miscarriage: a randomized controlled trial of expectant management versus surgical evacuation. Hum Reprod [Internet]. 2002;17(9):2445–50. Available from: http://humrep.oxfordjournals.org/lookup/doi/10.1093/humrep/17.9.2445. 12202439 10.1093/humrep/17.9.2445

[7] You JHS, Chung TKH. Expectant, medical or surgical treatment for spontaneous abortion in first trimester of pregnancy: A cost analysis. Hum Reprod. 2005;20(10):2873–8. doi: 10.1093/humrep/dei163 15979988

[8] Niinimäki M, Jouppila P, Martikainen H, Talvensaari-Mattila A. A randomized study comparing efficacy and patient satisfaction in medical or surgical treatment of miscarriage. Fertil Steril [Internet]. 2006;86(2 CC-Consumers and Communication CC-Pregnancy and Childbirth):367‐372. Available from: https://www.cochranelibrary.com/central/doi/10.1002/central/CN-00565786/full. 16764872 10.1016/j.fertnstert.2005.12.072

[9] Lemmers M, Verschoor MAC, Hooker AB, Opmeer BC, Limpens J, Huirne JAF, et al. Dilatation and curettage increases the risk of subsequent preterm birth: a systematic review and meta-analysis. Hum Reprod [Internet]. 2016;31(1):34–45. Available from: https://academic.oup.com/humrep/article-lookup/doi/10.1093/humrep/dev274. 26534897 10.1093/humrep/dev274

[10] ACOG. Clinical Management Guidelines for Obstetrician–Gynecologists. Obstet Gynecol. 2019;133(76):168–86. doi: 10.1097/AOG.0000000000003324 31241598

[11] Pfizer Limited. Cytotec UK SPC (Summary of Product Characteristics) [Internet]. Available from: https://www.medicines.org.uk/emc/product/1642/smpc.

[12] Fernlund A, Jokubkiene L, Sladkevicius P, Valentin L. Misoprostol treatment vs expectant management in women with early non-viable pregnancy and vaginal bleeding: a pragmatic randomized controlled trial. Ultrasound Obstet Gynecol [Internet]. 2018 Jan;51(1):24–32. Available from: http://ovidsp.ovid.com/ovidweb.cgi?T=JS&PAGE=reference&D=emexa&NEWS=N&AN=623940672. doi: 10.1002/uog.18940 29072372

[13] Bagratee JS, Khullar V, Regan L, Moodley J, Kagoro H. A randomized controlled trial comparing medical and expectant management of first trimester miscarriage. Hum Reprod. 2004;19(2):266–71. doi: 10.1093/humrep/deh049 14747165

[14] Graziosi GCM, Mol BWJ, Reuwer PJH, Drogtrop A, Bruinse HW. Misoprostol versus curettage in women with early pregnancy failure after initial expectant management: A randomized trial. Hum Reprod. 2004;19(8):1894–9. doi: 10.1093/humrep/deh344 15192065

[15] Van Den Berg J, Van Den Bent JM, Snijders MP, De Heus R, Coppus SF, Vandenbussche FP. Sequential use of mifepristone and misoprostol in treatment of early pregnancy failure appears more effective than misoprostol alone: A retrospective study. Eur J Obstet Gynecol Reprod Biol [Internet]. 2014;183:16–9. Available from: doi: 10.1016/j.ejogrb.2014.10.010 25461345

[16] Hamel C, Coppus S, van den Berg J, Hink E, van Seeters J, van Kesteren P, et al. Mifepristone followed by misoprostol compared with placebo followed by misoprostol as medical treatment for early pregnancy loss (the Triple M Trial): a double-blind placebo-controlled randomised trial. EClinicalMedicine. 2020;In press. 10.1016/j.eclinm.2020.100716 PMC7910666 33681738

[17] Schreiber CA, Creinin MD, Atrio J, Sonalkar S, Ratcliffe SJ, Barnhart KT. Mifepristone Pretreatment for the Medical Management of Early Pregnancy Loss. N Engl J Med [Internet]. 2018;378(23):2161–70. Available from: http://www.nejm.org/doi/10.1056/NEJMoa1715726. 29874535 10.1056/NEJMoa1715726 PMC6437668

[18] Chu JJ, Devall AJ, Beeson LE, Hardy P, Cheed V, Sun Y, et al. Mifepristone and misoprostol versus misoprostol alone for the management of missed miscarriage (MifeMiso): a randomised, double-blind, placebo-controlled trial. Lancet [Internet]. 2020;6736(20):1–9. Available from: doi: 10.1016/S0140-6736(20)31788-8 32853559 PMC7493715

[19] Van Den Berg J, Hamel CC, Snijders MP, Coppus SF, Vandenbussche FP. Mifepristone and misoprostol versus misoprostol alone for uterine evacuation after early pregnancy failure: Study protocol for a randomized double blinded placebo-controlled comparison (Triple M Trial). BMC Pregnancy Childbirth. 2019;19(1):1–8. doi: 10.1186/s12884-018-2145-y 31775677 PMC6880504

[20] Committee for Medicinal Products for Human Use (CHMP). Guideline on missing data in confirmatory clinical trials. London Eur Med Agency. 2010;44(July 2010):1–12.

[21] Brazier J, Roberts J, Deverill M. The estimation of a preference-based measure of health from the SF-36. J Health Econ. 2002;21(2):271–92. doi: 10.1016/s0167-6296(01)00130-8 11939242

[22] Elbasha EH, Chhatwal J. Myths and Misconceptions of Within-Cycle Correction: A Guide for Modelers and Decision Makers. Pharmacoeconomics. 2016;34(1):13–22. doi: 10.1007/s40273-015-0337-0 26643402

[23] Nagendra D, Koelper N, Loza-Avalos SE, Sonalkar S, Chen M, Atrio J, et al. Cost-effectiveness of Mifepristone Pretreatment for the Medical Management of Nonviable Early Pregnancy: Secondary Analysis of a Randomized Clinical Trial. JAMA Netw open. 2020;3(3):e201594. doi: 10.1001/jamanetworkopen.2020.1594 32215633 PMC7439768

[24] Berkley HH, Greene HL, Wittenberger MD. Mifepristone Combination Therapy Compared With Misoprostol Monotherapy for the Management of Miscarriage: A Cost-Effectiveness Analysis. Obstet Gynecol. 2020;136(4):774–81. doi: 10.1097/AOG.0000000000004063 32925621

[25] Milingos DS, Mathur M, Smith NC, Ashok PW. Manual vacuum aspiration: A safe alternative for the surgical management of early pregnancy loss. BJOG An Int J Obstet Gynaecol [Internet]. 2009;116(9):1268–71. Available from: http://ovidsp.ovid.com/ovidweb.cgi?T=JS&PAGE=reference&D=emed11&NEWS=N&AN=355060182. doi: 10.1111/j.1471-0528.2009.02223.x 19459859

[26] Tasnim N, Mahmud G, Fatima S, Sultana M. Manual vacuum aspiration: a safe and cost-effective substitute of electric vacuum aspiration for the surgical management of early pregnancy loss. J Pak Med Assoc [Internet]. 2011;61(2 CC-Pregnancy and Childbirth):149‐153. Available from: https://www.cochranelibrary.com/central/doi/10.1002/central/CN-00778646/full. 21375164

[27] Saurel-Cubizolles MJ, Opatowski M, David P, Bardy F, Dunbavand A. Pain during medical abortion: A multicenter study in France. Eur J Obstet Gynecol Reprod Biol. 2015;194:212–7. doi: 10.1016/j.ejogrb.2015.09.025 26448133

[28] Neumann PJ, Cohen JT, Weinstein MC. Updating cost-effectiveness—The curious resilience of the $50,000-per-QALY threshold. N Engl J Med. 2014;371(9):796–7. doi: 10.1056/NEJMp1405158 25162885

[29] Dutch Institute National Health Care (Zorginstituut Nederland). Richtlijn voor het uitvoeren van economische evaluaties in de gezondheidzorg (Protocol for the execution of economic evaluation in healthcare). 29-02-2016 [Internet]. 2016;(november):120. Available from: https://www.ispor.org/PEguidelines/source/NL-Economic_Evaluation_Guidelines.pdf.

[30] Hamel CC, van Wessel S, Carnegy A, Coppus SFPJ, Snijders MPML, Clark TJ, et al. Towards well-defined diagnostic criteria for retained products of conception (RPOC): a systematic scoping review. Manuscr Submitt Publ.

[31] Lemmers M, Verschoor MAC, Oude Rengerink K, Naaktgeboren C, Bossuyt PM, Huirne JAF, et al. MisoREST: Surgical versus expectant management in women with an incomplete evacuation of the uterus after misoprostol treatment for miscarriage: A cohort study. Hum Reprod. 2017;211:83–9. doi: 10.1016/j.ejogrb.2017.01.019 28209537

[32] Lemmers M, Verschoor MAC, Bossuyt PM, Huirne JAF, Spinder T, Nieboer TE, et al. Cost-effectiveness of curettage versus expectant management in women with an incomplete evacuation after misoprostol treatment for first-trimester miscarriage: a randomized controlled trial and cohort study. Obstet Gynecol Surv [Internet]. 2018;73(7):398‐399. Available from: https://www.cochranelibrary.com/central/doi/10.1002/central/CN-01629475/full. 10.1111/aogs.13283 29266169

[33] Lemmers M, Verschoor MAC, Broekhuijsen K, Rengerink OK, Opmeer BC, Bossuyt PM, et al. Quality of life for curettage versus expectant management in women with incomplete evacuation of the uterus after treatment with misoprostol for miscarriage: the misorest trial. Hum Reprod [Internet]. 2015;30:i185‐. Available from: https://www.cochranelibrary.com/central/doi/10.1002/central/CN-01098507/full.

