# Supplementary material for: Economic evaluation of a randomized controlled trial comparing mifepristone and misoprostol with misoprostol alone in the treatment of early pregnancy loss
Source: PLoS One. 2022 Feb 9;17(2):e0262894. doi: 10.1371/journal.pone.0262894 (PMC8827447; doi:10.1371/journal.pone.0262894)
Supplement: Attachment — Submitted filename: renamed_a5b17.docx [file pone.0262894.s001.docx]

**COMMENTS – Manuscript PONE-D-21-02362**

Title: “Economic evaluation of a randomized controlled trial comparing mifepristone and misoprostol with misoprostol alone in the treatment of early pregnancy loss”

**Important note: This review pertains only to ‘statistical aspects’ of the study and so** **‘clinical aspects’ [like medical importance, relevance of the study,** **‘clinical significance and implication(s)’ of the whole study, etc.] are to be evaluated [should be assessed] separately/independently. Further please note that any ‘statistical review’ is generally done under the assumption that (such) study specific methodological [as well as execution] issues are perfectly taken care of by the investigator(s). This review is not an exception to that and so does not cover clinical aspects {however, seldom comments are made only if those issues are intimately / scientifically related & intermingle with ‘statistical aspects’ of the study}. Agreed that ‘statistical methods’ are used as just tools here, however, they are vital part of methodology [and so should be given due importance].**

**COMMENTS:** In my opinion, it may be useful to add [for general / non-subject expert readers] that ‘Expectant management’ means waiting for the miscarriage to happen by itself naturally, without treatment]. Since misoprostol orally is common to both it is perfectly alright to administer ‘mifepristone 600mg (N=175)’ to one group and its ‘placebo (N=176)’ to other, however, more details of ‘placebo’ are missing [mention that ‘an identical appearing placebo’ may not suffice]. Moreover, your saying “medical treatment with placebo followed by misoprostol to be 26% more expensive compared to mifepristone followed by misoprostol (p=0.001)” is not very clear because the difference is ‘mifepristone 600mg’ versus its ‘placebo’ which implies that ‘placebo’ is more expensive [how medical fraternity or patients will benefit by such info?]. If you intend to convey something else, mention that clearly.

Description/details regarding many of the Important items in CONSORT checklist [Examples: Allocation concealment (item 9), Blinding (item 11a)] are not found. This trial is said to be ‘double-blinded placebo-controlled RCT’ but no blinding details included. The word ‘CONSORT’ itself is surprisingly not found in the article. The economic evaluation was performed correctly but there is hardly anything on other analytical {mainly statistical} methods used. Study protocol for this trial is published separately [BMC Pregnancy Childbirth. 2019;19(1):1–8] is good, however, few details about study design, sample size calculation, study procedures and outcome should have been briefly described here also. As you clarified, you are presenting a cost-effectiveness analysis (CEA), [performed as a secondary analysis], but note that this one is separate article (and not a part series), therefore, few details are expected/mandatory I guess.

Statistical comparison of baseline characteristics [last ‘p-value’ column in Table 1] is not desirable at all. In this context, read the following:

To provide a description of baseline characteristics is entirely reasonable (since it is clearly important in assessing to whom the results of the trial can be applied), however, it does not require the division of baseline characteristics by treatment groups (however, if done – alright). Statistical comparison of baseline characteristics is not desirable at all [because even if P-value turns out to be significant (while comparing baseline characteristics despite random allocation), it is, by definition, a false positive] as you then are supposed to be testing ‘randomization’ then, which in any single trial may not balance all baseline characteristics because ‘randomization’ is a sort of ‘insurance’ and not a guarantee scheme.

References:

1. Stuart J. Pocock, et al., ‘Subgroup analysis, covariate adjustment and baseline comparisons in clinical trial reporting: current practice and problems’, Statistics in medicine, 2002; 21:2917–2930 [Particularly page 2927]
2. Harrington D, et al., ‘New guidelines for statistical reporting in the journal’, N Engl J Med 2019;381:285-6

[Important message (indirectly/ultimately indicated) from these articles: Never do any comparison with respect to ‘baseline’ characteristics {by applying statistical significance test(s)}, when allocation is done randomly].

In last ‘p-value’ column in Table 1, there are three (or more) ‘n.s.’ appearing with respect to few variables/characteristics [ex.: Gravidity], which implies that each row is tested separately. Is that correct? Which test is applied for table-2 data? Though the measures/tools used are appropriate, most of them [including SF-36 {as you said: Quality Adjusted Life Years (QALY’s) were calculated from participants’ scores on the SF-36 questionnaires sent digitally at treatment start, and one, two and six weeks later} yield data that are in [at the most] ‘ordinal’ level of measurement [and not in ratio level of measurement for sure {as the score two times higher does not indicate presence of that parameter/phenomenon as double (for example, a Visual Analogue Scales VAS score or say ‘depression’ score)}]. Then application of suitable non-parametric test(s) is/are indicated/advisable [even if distribution may be ‘Gaussian’ (i.e. normal)]. Agreed that there is/are no non-parametric test(s)/technique(s) available to be used as alternative in all situation(s) [suitable / most desired/applicable], but should be used whenever/wherever they are available.

Since missing data were imputed using the ‘last observation carried forward’, I hope the authors are aware of disadvantages [like this method assumes that the response remains constant at the last observed value. This assumption can be biased if the timing and the rate of withdrawal is related to the treatment (e.g. in the case of degenerative diseases, using the last observed value to impute for missing data at a later point in the study means that a higher observation will be carried forward, resulting in an overestimation of the true end-of study measurement)] of the method (must be known to these learned authors, still may please be noted) that:

“according to available literature [example, “Inference and Missing Data,” Biometrika, 1976, vol:63, 581–592 and “Multiple Imputation After 18+ Years,” Journal of the American Statistical Association, 1996, vol:91, 473–489] ‘Multiple Imputation’ technique is preferred [considering MCAR (Missing Completely At Random) expected nature of data] than {despite being time-consuming and involving much more computations} compare to all out of other important imputation techniques frequently used [like Group Means, Hot-deck Imputation, Baseline Observation Carried Forward (BOCF), Worst Observation Carried Forward (WOCF), Predicted Mean, and even Last Observation Carried Forward (LOCF)].”

Except these minor points, the article is excellent [by all the means]. It is definitely in ‘acceptable’ category. Very good job. Appreciated.
