# Supplementary material for: Economic evaluation of a randomized controlled trial comparing mifepristone and misoprostol with misoprostol alone in the treatment of early pregnancy loss
Source: PLoS One. 2022 Feb 9;17(2):e0262894. doi: 10.1371/journal.pone.0262894 (PMC8827447; doi:10.1371/journal.pone.0262894)
Supplement: Attachment — Submitted filename: Response to reviewers Plos One def.docx [file pone.0262894.s002.docx]

**Reviewer #1:** This is a well conducted secondary analysis of the cost-effectiveness data for the Triple-M trial. This study was pre-registered and all outcomes were reported as specified. I only have one minor comment, which is that the last sentence of the discussion is incomplete 'Future dose-finding studies might'.

*We thank the reviewer for his/her efforts, and positive comments. Indeed, the last sentence of the discussion appears to have disappeared during the submission process. We have altered this in our revised manuscript (page 16, lines 339-340).*

**Reviewer #2:** Important note: This review pertains only to ‘statistical aspects’ of the study and so ‘clinical aspects’ [like medical importance, relevance of the study, ‘clinical significance and implication(s)’ of the whole study, etc.] are to be evaluated [should be assessed] separately/independently. Further please note that any ‘statistical review’ is generally done under the assumption that (such) study specific methodological [as well as execution] issues are perfectly taken care of by the investigator(s). This review is not an exception to that and so does not cover clinical aspects {however, seldom comments are made only if those issues are intimately / scientifically related & intermingle with ‘statistical aspects’ of the study}. Agreed that ‘statistical methods’ are used as just tools here, however, they are vital part of methodology [and so should be given due importance].

COMMENTS: In my opinion, it may be useful to add [for general / non-subject expert readers] that ‘Expectant management’ means waiting for the miscarriage to happen by itself naturally, without treatment]. Since misoprostol orally is common to both it is perfectly alright to administer ‘mifepristone 600mg (N=175)’ to one group and its ‘placebo (N=176)’ to other, however, more details of ‘placebo’ are missing [mention that ‘an identical appearing placebo’ may not suffice]. Moreover, your saying “medical treatment with placebo followed by misoprostol to be 26% more expensive compared to mifepristone followed by misoprostol (p=0.001)” is not very clear because the difference is ‘mifepristone 600mg’ versus its ‘placebo’ which implies that ‘placebo’ is more expensive [how medical fraternity or patients will benefit by such info?]. If you intend to convey something else, mention that clearly.

*We agree with the reviewers that for the general/non-subject readers, expectant management could be explained more thoroughly, and have done so in the manuscript on page 4, line 77.*

*Furthermore, we have provided more details about the placebo tablets (page 5, line 113) and elaborated on the average costs per patient in the placebo group versus those in the mifepristone group. We have clarified that although the placebo tablets itself are less expensive, average treatment costs are higher in those (pre)treated with placebo compared to those (pre)treated with mifepristone on page 9, lines 205-206..*

Description/details regarding many of the Important items in CONSORT checklist [Examples: Allocation concealment (item 9), Blinding (item 11a)] are not found. This trial is said to be ‘double-blinded placebo-controlled RCT’ but no blinding details included. The word ‘CONSORT’ itself is surprisingly not found in the article. The economic evaluation was performed correctly but there is hardly anything on other analytical {mainly statistical} methods used. Study protocol for this trial is published separately [BMC Pregnancy Childbirth. 2019;19(1):1–8] is good, however, few details about study design, sample size calculation, study procedures and outcome should have been briefly described here also. As you clarified, you are presenting a cost-effectiveness analysis (CEA), [performed as a secondary analysis], but note that this one is separate article (and not a part series), therefore, few details are expected/mandatory I guess.

*We have indeed not provided a CONSORT checklist, as the primary results of our trial have been published elsewhere. We refer to this paper published in EClinicalMedicine (https://www.thelancet.com/journals/eclinm/article/PIIS2589-5370(20)30460-0/fulltext)*

*in the manuscript (ref #19), which is accompanied by a CONSORT checklist and contains all details concerning allocation concealment/randomization/blinding etc.*

*As this manuscript focuses on the cost-effectiveness of pre-treatment with mifepristone, we deemed a more detailed description of our RCT was not appropriate here, we have however specifically mentioned in our manuscript that a CONSORT checklist can also be found in our prior publication of the primary outcomes of the Triple M Trial. (page 5, line 107).*

Statistical comparison of baseline characteristics [last ‘p-value’ column in Table 1] is not desirable at all. In this context, read the following:
To provide a description of baseline characteristics is entirely reasonable (since it is clearly important in assessing to whom the results of the trial can be applied), however, it does not require the division of baseline characteristics by treatment groups (however, if done – alright). Statistical comparison of baseline characteristics is not desirable at all [because even if P-value turns out to be significant (while comparing baseline characteristics despite random allocation), it is, by definition, a false positive] as you then are supposed to be testing ‘randomization’ then, which in any single trial may not balance all baseline characteristics because ‘randomization’ is a sort of ‘insurance’ and not a guarantee scheme.
References:
1. Stuart J. Pocock, et al., ‘Subgroup analysis, covariate adjustment and baseline comparisons in clinical trial reporting: current practice and problems’, Statistics in medicine, 2002; 21:2917–2930 [Particularly page 2927]
2. Harrington D, et al., ‘New guidelines for statistical reporting in the journal’, N Engl J Med 2019;381:285-6
[Important message (indirectly/ultimately indicated) from these articles: Never do any comparison with respect to ‘baseline’ characteristics {by applying statistical significance test(s)}, when allocation is done randomly].

In last ‘p-value’ column in Table 1, there are three (or more) ‘n.s.’ appearing with respect to few variables/characteristics [ex.: Gravidity], which implies that each row is tested separately. Is that correct? Which test is applied for table-2 data? Though the measures/tools used are appropriate, most of them [including SF-36 {as you said: Quality Adjusted Life Years (QALY’s) were calculated from participants’ scores on the SF-36 questionnaires sent digitally at treatment start, and one, two and six weeks later} yield data that are in [at the most] ‘ordinal’ level of measurement [and not in ratio level of measurement for sure {as the score two times higher does not indicate presence of that parameter/phenomenon as double (for example, a Visual Analogue Scales VAS score or say ‘depression’ score)}]. Then application of suitable non-parametric test(s) is/are indicated/advisable [even if distribution may be ‘Gaussian’ (i.e. normal)]. Agreed that there is/are no non-parametric test(s)/technique(s) available to be used as alternative in all situation(s) [suitable / most desired/applicable], but should be used whenever/wherever they are available.

*The baseline characteristics were added to show that differences between both treatment groups regarding cost-effectiveness cannot be attributed to anything else but the treatment. However, as it is not deemed appropriate by this reviewer, we adjusted the manuscript accordingly, and have deleted the p-values from table 1. For table 2, comparing means of the study groups, a T-test was used.*

Since missing data were imputed using the ‘last observation carried forward’, I hope the authors are aware of disadvantages [like this method assumes that the response remains constant at the last observed value. This assumption can be biased if the timing and the rate of withdrawal is related to the treatment (e.g. in the case of degenerative diseases, using the last observed value to impute for missing data at a later point in the study means that a higher observation will be carried forward, resulting in an overestimation of the true end-of study measurement)] of the method (must be known to these learned authors, still may please be noted) that:
“according to available literature [example, “Inference and Missing Data,” Biometrika, 1976, vol:63, 581–592 and “Multiple Imputation After 18+ Years,” Journal of the American Statistical Association, 1996, vol:91, 473–489] ‘Multiple Imputation’ technique is preferred [considering MCAR (Missing Completely At Random) expected nature of data] than {despite being time-consuming and involving much more computations} compare to all out of other important imputation techniques frequently used [like Group Means, Hot-deck Imputation, Baseline Observation Carried Forward (BOCF), Worst Observation Carried Forward (WOCF), Predicted Mean, and even Last Observation Carried Forward (LOCF)].”

*We acknowledge that there are other imputation methods less prone to bias and that last observation carried forward is only unbiased when the distribution of the observed values at time ‘t’ is exactly equal to the distribution of the missing values at time t+1. However, LOCF is a straight forward missing value imputation method, it improves power and allows the analysis to examine trends over time. Given the fact that the pattern over time in the experimental and control group is pretty much the same (before and after imputation) it does not bias (in a conservative way) the experimental group compared to the control. Also, the trend observed after LOCF is similar to that in the pilot study of our RCT. All this gives sufficient confidence in the analysis including LOCF.*

*We will mention this in the discussion.*

Except these minor points, the article is excellent [by all the means]. It is definitely in ‘acceptable’ category. Very good job. Appreciated.

*We thank the reviewer for his/her positive words, and comments improving our manuscript.*

Reviewer #3: This is a well presented manuscript presenting the cost effectiveness analysis of a robustly performed multi-centre randomised controlled trial - Triple M - from the Netherlands. The trial was curtailed prematurely due to the findings of the interim data analysis due to clear benefit seen in women receiving mifepristone in combination with misoprostol in the medical management of early pregnancy loss (EPL).

This study shows that mifepristone and misoprostol incurs a significantly lower cost when compared to misoprostol alone. A cost saving of 26%. The authors have demonstrated that the main reasons for reducing cost if a lower cost of medications, admissions and a reduced need for uterine aspiration.

There are some improvements that could be made to the manuscript though:

I would suggest that the editorial board review the manuscript to aid the authors improve English language. There are grammatical and stylistic issues throughout the manuscript. There are also some scientific writing issues in the manuscript such as the use of abbreviations within the abstract of the manuscript and also the use of a question within the background of the abstract. Another example, would be the abbreviation of EPL at the beginning of the abstract which had not yet been explained.

*We thank the reviewer for his/her suggestion, and have altered the manuscript, making sure all abbreviations are explained prior to further use in the manuscript, and grammatical and stylistic issues are solved.*

The introduction is excellent and sets the scene very well. The authors have acknowledged the other trials and cost-effectiveness analyses performed on this research question that have been published before.

The method section is well written and the tools used for economic evaluation are well described and well established in health economic evaluation research.

The results section demonstrate the cost benefits clearly and the fact that there are no real differences in the two trial arms when measuring societal costs.

The discussion makes appropriate deductions from the data provided in the results section. The end of the discussion does seem to be incomplete and the authors will need to rectify this.

*As also mentioned by reviewer #1, indeed, the last sentence of the discussion appears to have disappeared during the submission process. We have altered this in our revised manuscript. (page 16, lines 339-340).*
